# Glucocorticoid effects in the regenerating fin reflect tissue homeostasis disturbances in zebrafish by affecting Wnt signaling

**DOI:** 10.3389/fendo.2023.1122351

**Published:** 2023-05-29

**Authors:** Lisa Fleischhauer, Alejandra Cristina López-Delgado, Karina Geurtzen, Franziska Knopf

**Affiliations:** ^1^ CRTD – Center for Regenerative Therapies, TU Dresden, Dresden, Germany; ^2^ Center for Healthy Aging, Faculty of Medicine Carl Gustav Carus TU Dresden, Dresden, Germany; ^3^ Laboratory of Clinical and Experimental Endocrinology, Department of Chronic Diseases, Metabolism and Ageing, KU Leuven, Leuven, Belgium

**Keywords:** glucocorticoid, zebrafish, fin regeneration, Wnt signaling, cell proliferation, skin, intestine, goblet cell

## Abstract

As a treatment for various immune-mediated diseases, the use of glucocorticoids as anti-inflammatory and immunosuppressive agents is common practice. However, their use is severely hampered by the risk of the development of adverse effects such as secondary osteoporosis, skin atrophy, and peptic ulcer formation. The exact molecular and cellular mechanisms underlying those adverse effects, which involve most major organ systems, are not yet fully understood. Therefore, their investigation is of great importance to improve treatment regimens for patients. Here, we investigated the effects of the glucocorticoid prednisolone on cell proliferation and Wnt signaling in homeostatic skin and intestinal tissue and compared them to the anti-regenerative effects in zebrafish fin regeneration. We also investigated a potential recovery from the glucocorticoid treatment and the impact of short-term treatment with prednisolone. We identified a dampening effect of prednisolone on Wnt signaling and proliferation in highly proliferative tissues, namely the skin and intestine, as well as reduced fin regenerate length and Wnt reporter activity in the fin. The presence of the Wnt inhibitor Dickkopf1 was enhanced in prednisolone treated skin tissue. A decreased number of mucous producing goblet cells was observed in the intestine of prednisolone treated zebrafish. Unexpectedly, proliferation in bone forming osteoblasts of the skull, homeostatic scales, as well as the brain was not decreased, opposite to the observed effects in the skin, fin, and intestine. Short-term treatment with prednisolone for a few days did not significantly alter fin regenerate length, skin cell proliferation, intestinal leukocyte number and proliferation of intestinal crypt cells. However, it affected the number of mucous-producing goblet cells in the gut. Likewise, discontinuation of prednisolone treatment for a few days saved the skin and intestine from a significant reduction of skin and intestinal cell proliferation, intestinal leukocyte number and regenerate length, but did not rescue goblet cell number. The suppressive effects of glucocorticoids in highly proliferative tissues may be relevant in the context of their therapeutic applications in patients with inflammatory diseases.

## 1 Introduction

Glucocorticoids (GCs) are a group of commonly used potent therapeutic agents to treat immune-mediated diseases in humans, because of their anti-inflammatory and immunosuppressive functions. Due to their lipophilic character, GCs can freely diffuse through the cell membrane and directly bind to the glucocorticoid receptor (GR) located in the cells’ cytoplasm. Upon binding to the GR, GCs lead to a conformational change and the translocation of the GR into the nucleus. In the nucleus, the GR either interacts with other DNA-bound transcription factors or directly binds to DNA sequences known as GC response elements to exert the signaling molecule’s function (1). *Via* the GR-DNA interaction, GCs increase gene expression of anti-inflammatory proteins such as Lipocortin-1, Interleukin-1 receptor, and Interleukin-10 (2). Depending on the type and physiological state of the cell, the GR can regulate many physiological functions including energy homeostasis, embryonic and postembryonic development, and the stress response (3). GCs show several adverse effects that involve most major organ systems. In particular, long-term use of GCs is associated with a rapid and pronounced decrease in bone mineral density and an increased fracture risk. This undesired consequence is a common form of secondary osteoporosis and is known as GC-induced osteoporosis (4). Depending on their potency and the duration of the therapy, GCs have been shown to be an inducer of numerous cutaneous side effects. Skin atrophy is the most frequent side effect of long-term topical treatment, by which the skin becomes thin and fragile. Atrophy of the skin affects all parts of the skin and results in increased permeability of the stratum corneum barrier, the outermost layer of the epidermis, accounting for increased transepidermal water loss (2). This effect was reported to be a result of GCs suppressing cutaneous cell proliferation and protein synthesis in particular of keratinocytes and dermal fibroblasts (5). Ultimately, GC treatment can result in irreversible stretch marks and disturbed wound healing (2). Furthermore, the use of GCs in combination with non-steroidal anti-inflammatory drugs is associated with a twofold increase in the risk of gastrointestinal adverse reactions among patients and a significant rise in the risk of developing peptic ulcer disease (6). Nevertheless, GCs are a regular treatment option for patients with inflammatory intestinal diseases such as Bowel’s disease (7).

Zebrafish have become an important model organism to study vertebrate development, mimic human diseases and investigate complex tissue regeneration, the latter due to their spectacular capability to regenerate various organs even after extensive trauma (8, 9). Genetic and chemical screens can be performed at low cost, and the identification and investigation of gene function in zebrafish is feasible because of their genetic amenability. The amputated part of the zebrafish fin is readily replaced and therefore provides a valuable model to study regeneration (10). Previously, effects of sustained GC-treatment on bone regeneration have been investigated by using the zebrafish fin, skull and spine. Rapid anti-inflammatory, bone inhibitory and anti-regenerative effects were observed upon treatment with the synthetic GC prednisolone in injured bone. These effects were characterized by a reduction of innate immune cells (macrophages), reduced fin regenerate length, impaired osteoblast (bone matrix forming cell) proliferation and their diminished differentiation (11). Anti-osteogenic effects were discovered in tissues undergoing high rates of proliferation, while bone volume was unaltered in the uninjured spine after a 2-month GC treatment (11). Studies on mice documented an induction of autophagy and partially even apoptosis of osteoblasts and terminally differentiated osteoblasts (osteocytes) in the presence of GCs (12, 13). Furthermore, a continued reduction in osteoblast activity subsequent to an elevation in osteoclast activity, as well as an increase of Wnt/β-catenin antagonists such as Dickkopf1 and Sclerostin were observed upon GC treatment (14, 15). Expression of Wnt inhibitor genes by osteocytes during the exposure with GC were identified (12, 13).

Wnt signaling is a signaling pathway that plays a key role in regulating proliferation and differentiation in a wide variety of cell types. This signaling pathway can be classified into i) β-catenin-dependent canonical Wnt signaling and ii) β-catenin-independent non-canonical Wnt signaling. Canonical Wnt/β-catenin signaling regulates gene expression very early during the fin regeneration process and plays an important role in blastemal cell proliferation (16, 17). In the mammalian intestine and skin, Wnt signaling is a crucial regulator of organ development and adult homeostasis. It controls epidermal cell fate specification, morphogenesis, and hair follicle induction during skin development in mammals and is crucial for scale development and regeneration in teleost fish (18, 19). Later on, it regulates epidermal stem cell activation, maintenance and fate determination (19). At the base of mammalian intestinal crypts, rapidly cycling stem cells are marked by the expression of LGR5, which is a receptor that enhances Wnt/β-catenin signaling. LGR5+ cells are necessary for intestinal homeostasis by continuously generating new cells of the intestinal epithelium and Wnt signaling is essential for this process to happen (20). In clinical studies focusing on patients receiving initial GC therapy to treat systemic autoimmune disease, an increase of Wnt co-receptor inhibitors in the early phase of therapy along with a subsequent reduction of the ratio of Wnt to Wnt-ligand inhibitors could be observed in the serum, suggesting a suppressive effect of GCs on Wnt/β-catenin signaling leading to impaired bone formation (15, 21).

The above findings suggest that side effects of GC treatment on bone, skin and intestinal tissue might result from impaired cell proliferation, differentiation, and deprived Wnt signaling. However, the effects that GCs exert on Wnt signaling and cell proliferation beyond bone tissue in zebrafish are not well understood. Moreover, the reversibility of putative inhibitory effects on the above tissues is not yet described. This study aimed to close this knowledge gap by examining Wnt signaling and cell proliferation in the zebrafish skin, intestine and bone tissue in conjunction with fin regenerates undergoing GC treatment. Immunohistochemistry was performed on zebrafish tissues of individuals carrying a Wnt signaling reporter that had been exposed to high dose prednisolone treatment (22). In order to test for the reversibility of Wnt- and proliferation suppressive effects GC treatment was discontinued in some specimens. Proliferation and the intensity of the Wnt signaling reporter were used as readouts for tissue growth. To investigate the impact of prednisolone on proliferation and Wnt signaling in injured tissue, an assessment of treatment effects on fin regeneration was included, by examining regenerate length and Wnt activity in the fin’s proliferation zone. The resulting data show that reduced Wnt activity and regenerate length correlate well with defects in intestinal organ proliferation and reduced Wnt signaling in the skin.

## 2 Methods

### 2.1 Transgenic fish lines

For all the performed immunohistochemical experiments the transgenic zebrafish line Tg(*7xTCF-XIa.Siam*:nlsmCherry)^ia5^ (22) was used as a Wnt reporter line. In these zebrafish, the expression of the monomeric Cherry protein (mCherry) is under the control of seven multimerized TCF responsive elements upstream of the minimal promoter of the *Xenopus laevis* direct β-catenin target gene *siamois*, leading to mCherry fluorescence in Wnt signaling+ cells (22). mRNA used for RT-PCR expression analysis of Wnt/Notch/Fgf target genes and *dickkopf1b* was isolated from a different transgenic reporter zebrafish line [Tg(*Ola.Sp7:NLS-GFP*)^zf132^;Tg(*mpeg*:mCherry)^gl23^] (23, 24).

### 2.2 Fish husbandry, fin clipping and GC treatment

All procedures were performed in accordance with the animal handling and research regulations of the Landesdirektion Sachsen (permit numbers AZ DD25-5131/354/87, DD25-5131/450/4, 25-5131/496/56 and amendments). Fish were bred and maintained as described (25). The caudal fin of the zebrafish was clipped and left to regenerate under different treatment conditions. Directly after amputation zebrafish were either treated for 21 days with dimethylsulfoxide (DMSO) or 50 µM prednisolone as described previously (11, 26). In two other groups zebrafish were treated for 17 days with DMSO followed by 4 days of prednisolone treatment (short-term treatment) or alternatively for 17 days with prednisolone followed by 4 days of DMSO treatment (recovery). To isolate mRNA from regenerating caudal fins, treatment was performed for 7 days starting directly after amputation.

### 2.3 Imaging of fin regenerates

Regeneration of the fin was imaged in 0.02% MS222 (Merck)-anaesthetized individuals with a Zeiss SteREO Discovery.V12 stereomicroscope equipped with a AxioCam MRm and AxioVison software version 4.7.1.0. at 9, 14, 17, and 21 days post amputation (dpa), respectively. Imaging was reduced to a minimum of time points to ensure low experimental burden to the animals and started at 9 dpa as suppression of fin regenerate growth by prednisolone is robust at this stage (11).

### 2.4 Immunohistochemistry

#### 2.4.1 Fixation and decalcification of zebrafish

Zebrafish were euthanized at 21 dpa by using 0.1% MS222 and head and trunk regions were separately fixed in 4% paraformaldehyde (PFA, Electron Microscopy Sciences, 15710) in phosphate buffered saline (PBS) at 4°C overnight. The next day, specimens were transferred to a 1:1 mix of 4% PFA/PBS and Osteosoft (Merck) for one day. Thereafter, samples were transferred to Osteosoft for one additional day, after which they were transferred to PBS at 4°C for a 4-day wash. PBS was then exchanged with fresh PBS and remained in it for another three days, after which paraffin embedding was performed.

#### 2.4.2 Paraffin embedding and sectioning

Histological slides of the zebrafish tissues embedded in paraffin were generated by the histology facility of the CMCB (Center for Molecular and Cellular Bioengineering, Susanne Weiche and colleagues). First, the samples were dehydrated and infiltrated by using the 13:45 hour-program in the Microm STP 420D Tissue Processor (Thermo Fisher Scientific™ Inc.). Then, the samples were washed for 2 minutes with distilled water before they were dehydrated in an ascending ethanol (EtOH) series (45 minutes 40% EtOH, 45 minutes 70% EtOH, three times 60 minutes 96% EtOH, two times 60 minutes 100% EtOH). Finally, the samples were incubated twice in xylene for 50 minutes and infiltrated with Paraffin for four rounds for 60 minutes each time.

Paraffin-embedded zebrafish slides (1 µm transverse head sections, 2 µm sagittal trunk sections) at different stages (9, 14, 17 and 21 dpa) were generated with the help of the Rotary Microtome Microm HM 355 S (Thermo Fisher Scientific™ Inc.) by the histology facility of the CMCB (Susanne Weiche and colleagues).

#### 2.4.3 Rehydration of paraffin-embedded tissue slides

Paraffin slides were immersed in xylene (Carl Roth) two times for 10 minutes to remove paraffin from the tissue sections. Successively, xylene was removed by immersion in a decreasing EtOH-series (100%, 95%, 70%, 50%) twice in 100% EtOH for 10 minutes followed by lower concentrations of EtOH once for 5 minutes each (Fisher Chemical). After a final rinse in deionized water (dH_2_O), the samples were rehydrated in wash buffer (1xPBS) for 10 minutes.

#### 2.4.4 Antigen retrieval

Antigen retrieval was performed on slides before adding the primary antibody mix of dsRed, Proliferating-Cell-Nuclear-Antigen (PCNA) and Phospho-Histone H3 (PH3). Similarly, antigen retrieval was carried out before immunohistochemistry with Dickkopf1 (Dkk1) antibody. Staining for L-plastin was completed without antigen retrieval. Antigen retrieval was performed with the help of the histology facility of the CMCB (Susanne Weiche). After rehydration, slides were put into a heat cycler with 10 mM sodium citrate pH6. The samples were gradually heated up to 110°C in a DC-Module (Zytomed) and kept for 5 minutes at this temperature. Following this, the slides were left in the cycler for 30 minutes to cool down until immunohistochemistry was performed.

#### 2.4.5 Blocking, antibody treatment and DAPI staining

The rehydrated, and antigen retrieved (in case for dsRed, PCNA and PH3), tissue sections were incubated in blocking buffer (10% NGS, normal goat serum, Gibco, 16210-064) for 30 minutes at room temperature (RT), to prevent unspecific binding of the primary antibody to the samples. Following this, the tissue sections were incubated with primary antibody mix against dsRed (rabbit, polyclonal, 1:500, Clontech, 632496), PH3 (rabbit, polyclonal, 1:400, Merck Millipore, 06-570), and PCNA (mouse, IgG2a, 1:1000, Dako, M087901-2) or with a primary antibody against L-plastin (rabbit, polyclonal, 1:500, courtesy of Michael Brand) or Dkk1 (rabbit, 1:435, aviva systems biology, San Diego, USA, ARP55048_P050, with reactivity to zebrafish Dkk1 protein according to the manufacturer, raised against the following amino acids of the human DKK1 protein: CARHFWSKICKPVLKEGQVCTKHRRKGSHGLEIFQRCYCGEGLSCRIQKD) alone, diluted in blocking buffer at 2-8°C overnight. The next day, the surplus primary antibody was removed by washing the slides 3 times for 15 minutes with washing buffer (1xPBS). Secondary antibodies Alexa Fluor 555 goat anti-rabbit (1:800, Invitrogen, A21428) and Alexa Fluor 488 goat anti-mouse (1:800, Invitrogen, A11029) were diluted in 1xPBS and applied for 60 minutes at RT. Again, the slides were washed 3 times for 15 minutes with washing buffer (1xPBS), to remove the surplus antibody. Lastly, DAPI (4’,6-Diamidino-2-phenylindol, 1:5000 of 1 mg/ml stock in 1xPBS) was added to the slides and the samples were incubated for 2-5 minutes at RT, to stain cell nuclei. The slides were rinsed once with 1xPBS and mounted with an anti-fade mounting media (Vectashield^®^). Slides were stored at 2-8°C until visualization with the widefield fluorescence microscope.

### 2.5 Imaging of tissue sections, image analyses, and image processing

Images were obtained using an inverted Zeiss Axio Imager (widefield fluorescence mode) at a magnification of 40X. Mitotic nuclei were detected very rarely which is why PCNA was solely used as a readout for cell proliferation. This is in line with the fact that many more cells express PCNA during the cell cycle than PH3, which is only detectable during mitosis (27, 28). Images were acquired using identical settings for different groups. Image contrast, brightness, exposure and blacklevel were adjusted using the same settings across samples using GNU Image Manipulation Program (GIMP, GIMP 2.10.22). Intensity measurements of the mCherry signal in fin and skin were conducted using the Plot Profile Tool in Image J/Fiji Software. In the fin, the intensity was measured along the second, third and fourth fin ray of 5 to 8 individuals per group. In all other analyses of the skin, intestine, brain and skull, 5 individuals were used per group. Both sexes of the same age and housing conditions were used, with a maximum of 25% males per experimental group. We did not investigate sex-specific differences in our analyses. For the assessment of PCNA, Dkk1 and mCherry signal in the skin, different skin regions of the zebrafish head were assessed. The most representative regions of the data were chosen for figures. For measurements of mCherry signal in the skin, the plot profile tool was used on maximum width of 300 in Image J/Fiji Software (ImageJ 2.3.0/1.53q). Intensity was measured on three different areas per image starting from the outermost layer of the skin and proceeding inwards. First, two sections were analyzed each, with three measurements per section. From this, one fish average was calculated and used for the statistical analysis. Proliferating cell quantification and leukocyte quantifications was performed using the Image J/Fiji (ImageJ 2.3.0/1.53q) cell counter tool. PCNA+ cells were counted per microscopic image, if not stated otherwise. Overlay of different channel images was performed using Image J/Fiji Software (ImageJ 2.3.0/1.53q) and images were arranged with GIMP. The scheme displaying scale anatomy was created using DrawboardPDF (Drawboard 6.36.34.0).

### 2.6 RT-PCR

RNA samples were generated from fins at 7 dpa treated with DMSO or Prednisolone from 0 to 7 dpa using Trizol as described (11). cDNA was synthetized with the Transcriptor First Strand cDNA Synthesis Kit (Roche, 04897030001) according to the manufacturer’s instructions. RT-PCR was performed using DreamTaq Green DNA-Polymerase (Thermo Scientific, EP0713). Thermal cycling conditions recommended by the supplier were followed and 35 cycles were used. Primers used were the following (5’-3’): *fosl1b* Fw CCAGTGGTTTCTGCAGTCTT, *fosl1b* Rv AGCTGCTACCCTGTTCCTTT (amplicon size 244 bp), *dkk1b* Fw AATGACCCTGACATGATTCAGC, *dkk1b* Rv AGGCTTGCAGATTTTGGACC (amplicon size 213 bp) (29), *etv5b* Fw CGTTACAATGAGCAGGGTGT, *etv5b* Rv CGTCATACCCAAAACCCTCA (amplicon size 181 bp), *her6* Fw AGCTGCATGACACAGATCAA, *her6* Rv AGCTGGAAACCCCCATATAC (amplicon size 212 bp), *actb* Fw TTCACCACCACAGCCGAAAGA, *actb* Rv TACCGCAAGATTCCATACCCA (amplicon size 223 bp). We quantified *dkk1b* expression as this orthologue of human DKK1 has previously been shown to be expressed in regenerating zebrafish fins (17).

### 2.7 Statistical analyses

Graphpad Prism 9.2.0 for Windows 10 (Graphpad Software, La Jolla California) and R-studio (RStudio 2022.02.1 + 461) were used for statistical analysis and data visualization. Sampled data was tested for normal distribution using the Shapiro-Wilk test. Subsequently, normally distributed data was analyzed by parametric testing, using One-way ANOVA followed by a *post-hoc* Dunnett’s multiple comparison test or a two-tailed t-test. Data which did not follow a normal distribution was analyzed by non-parametric testing, using the Kruskal-Wallis test and *post hoc* Dunn’s multiple comparison. P-values ≤ 0.05 were considered statistically significant. Data were expressed as mean ± SEM (Standard Error of the Mean) for plot profiles and as mean ± SD (Standard Deviation) for all other graphs. We calculated group averages and SEM/SD based on the number of used individuals in the respective groups in order to display inter-individual variation. The respective required fish averages were calculated from a minimum of 2 sections and 3 fin rays, respectively, per individual. In case of PCNA+ skull osteoblast quantification, 1 section per individual was used; for PCNA+ scale forming cell quantification 6 scales on one section were analyzed per individual For details, see figure legends.

## 3 Results

### 3.1 GC treatment decreases fin regenerate length and affects Wnt signaling in the growth zone of the fin

**Figure 1:**
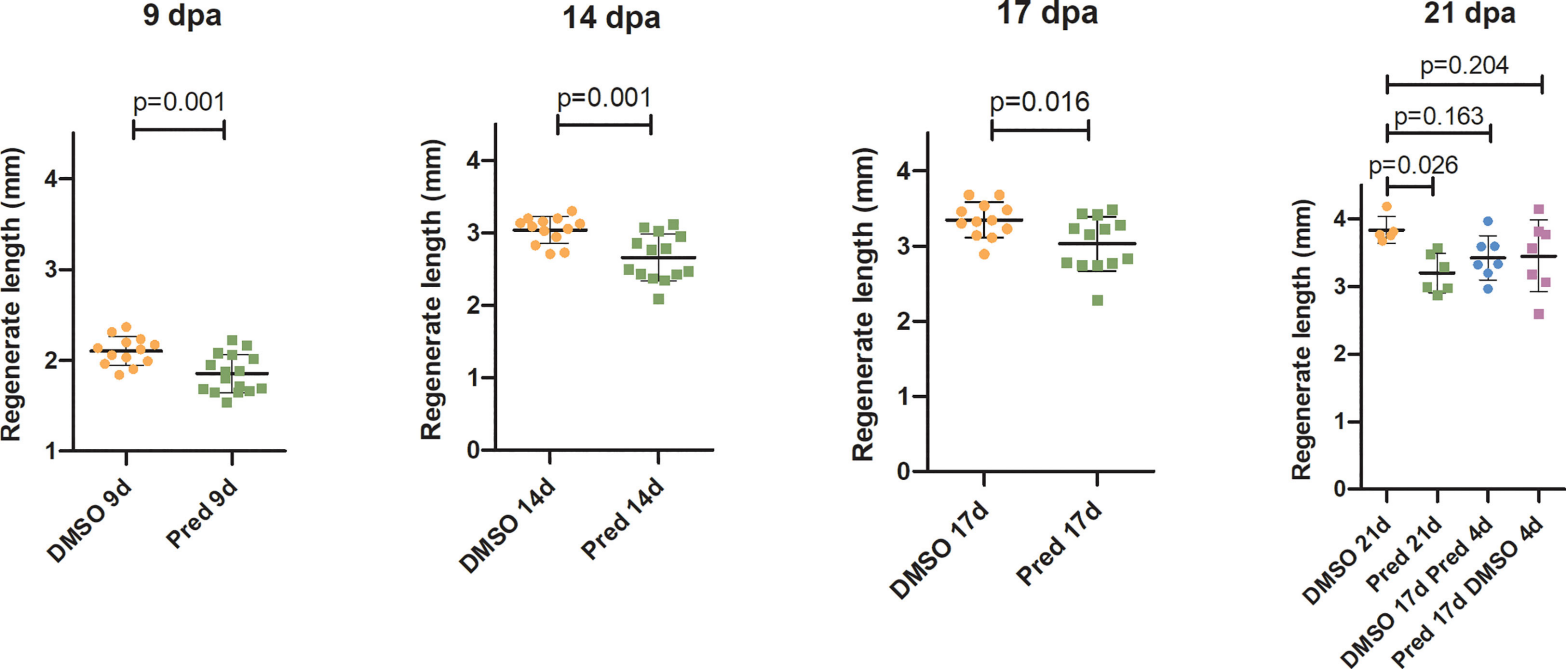
Quantification of fin regenerate length during the course of regeneration. Bars represent mean ± SD of fin outgrowth after amputation under different treatment conditions, measured at different times post amputation. Each dot represents one biological replicate. Parametric testing because of normal distribution of data. Statistical significance at 9 dpa, 14, dpa and 17 dpa was tested by two-tailed t-tests and at 21 dpa by *post-hoc* Dunnett’s multiple comparison after one-way ANOVA. n ^(DMSO,9dpa)^ = 13 (10 females, 3 males), n ^(Pred,9dpa)^ = 16 (12 females, 4 males), n ^(DMSO,14dpa)^ = 13 (10 females, 3 males), n ^(Pred,14dpa)^ = 14 (12 females, 2 males), n ^(DMSO,17dpa)^ = 12 (10 females, 2 males), n ^(Pred,17dpa)^ = 13 (12 females, 1 male), n ^(DMSO 21d,21dpa)^ = 5 (4 females, 1 male), n ^(Pred 21d,21dpa)^ = 6 (6 females), n ^(DMSO 17d Pred 4d,21dpa)^ = 7 (6 females, 1 male), n ^(Pred 17d DMSO 4d,21dpa)^ = 7 (6 females, 1 male).

The effect of prednisolone on regeneration and cell proliferation was analyzed by assessing fin regenerate length and Wnt signaling activity in the fin regenerate. Zebrafish were treated for 17 days post amputation (dpa) with prednisolone or DMSO as a control. In order to determine a possible recovery from prednisolone treatment, treatment with prednisolone was stopped at 17 dpa in a subset of zebrafish which were then incubated for 4 days with DMSO. In another subset of zebrafish, a 4-day prednisolone treatment was performed on previously DMSO treated zebrafish from 17 dpa onwards, in order to test for the impact of a short-term treatment during the late regeneration phase. This treatment regime was chosen to allow for sustained suppression of regeneration in terms of regenerate length with growth being stalled in prednisolone treated zebrafish starting at 17 dpa (11). Furthermore, with a total time of 21 days, the experimental burden on animals was reduced to a minimum, considering that longer treatments do not enhance anti-regenerative effects any further [see Figure 1H in (11)]. Regenerate length was measured at 9, 14, 17, and 21 dpa (**Figure 1**). For all treatments, an increase in regenerate length over time could be observed. Exclusive prednisolone treatment led to a significant reduction in the regenerate length compared to the control DMSO treatment at all-time points. While the fins of zebrafish treated with DMSO regenerated about 1700 µm, fins of prednisolone-treated zebrafish only regenerated 1300 µm within 12 days (from 9 dpa to 21 dpa). Discontinuation of prednisolone treatment after 17 days had a similar effect as prednisolone treatment during the last four days of the experiment; however, it did not significantly alter regenerate length in comparison to the 21-day DMSO control treatment (**Figure 1**).

**Figure 2:**
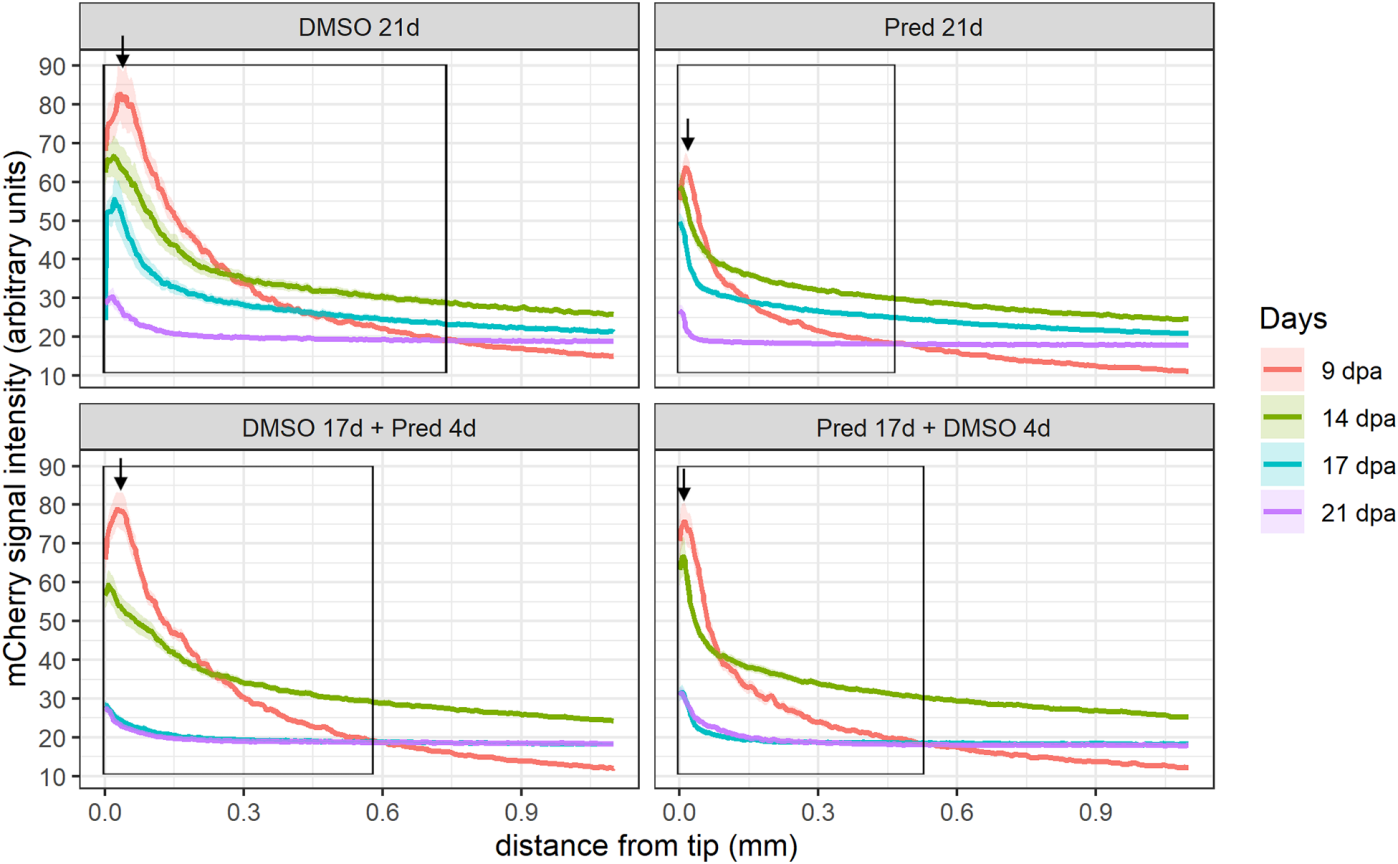
Quantification of the *7xTCF-Xia.Siam*:nlsmCherry Wnt reporter expression in the fin regenerate. Lines represent mean ± SEM of mCherry signal intensity for the four different treatments measured at 9 dpa, 14 dpa, 17 dpa, and 21 dpa. Signal was measured from the tip towards the amputation plane. Boxes indicate “Wnt-active zone” at 9 dpa, defined as levels beyond baseline expression levels detected at 21 dpa (i.e. point of intersection of 9 dpa curve with 21 dpa curve). n ^(DMSO 21d,9/14/17/21dpa)^ = 5 (4 females, 1 male); n ^(Pred 21d,9dpa)^ = 8 (6 females, 2 males), n ^(Pred 21d,14dpa)^ = 7 (6 females, 1 male), n ^(Pred 21d,17/21dpa)^ = 6 (6 females); n ^(DMSO 17d Pred 4d,9/14dpa)^ = 8 (6 females, 2 males), n ^(DMSO 17d Pred 4d,17/21dpa)^ = 7 (6 females, 1 male); n ^(Pred 17d DMSO 4d,9dpa)^ = 8 (6 females, 2 males), n ^(Pred 17d DMSO 4d,14/17/21dpa)^ = 7 (6 females, 1 male).

**Figure 3:**
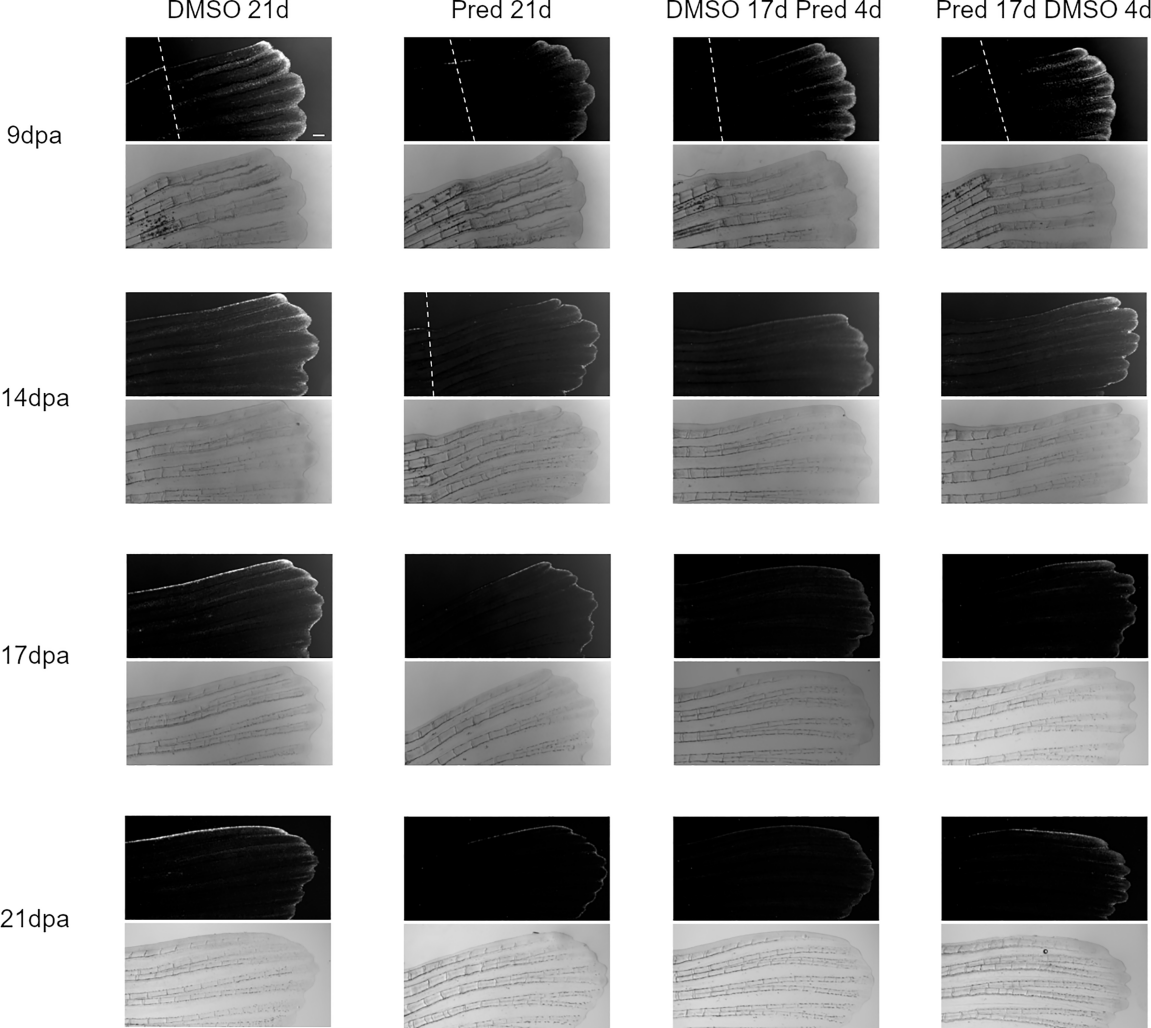
Images of the mCherry-fluorescent Wnt-active zone in zebrafish fin regenerates. The *7xTCF-Xia.Siam:*nlsmCherry expression is detectable due to mCherry fluorescence (upper rows). Corresponding brightfield images are shown below. Within one treatment group, the same individual is shown across time points. From left to right: fish underwent 21 days of DMSO, 21 days of prednisolone, 17 days of DMSO followed by 4 days of prednisolone and 17 days of prednisolone followed by 4 days of DMSO treatment. Scalebar 200 µm. The white dotted lines indicate amputation planes in the fluorescence view of the fins. n ^(DMSO 21d,9/14/17/21dpa)^ = 5 (4 females, 1 male); n ^(Pred 21d,9dpa)^ = 8 (6 females, 2 males), n ^(Pred 21d,14dpa)^ = 7 (6 females, 1 male), n ^(Pred 21d,17/21dpa)^ = 6 (6 females); n ^(DMSO 17d Pred 4d,9/14dpa)^ = 8 (6 females, 2 males), n ^(DMSO 17d Pred 4d,17/21dpa)^ = 7 (6 females, 1 male); n ^(Pred 17d DMSO 4d,9dpa)^ = 8 (6 females, 2 males), n ^(Pred 17d DMSO 4d,14/17/21dpa)^ = 7 (6 females, 1 male).

Next, the effect of prednisolone on Wnt signaling was assessed by quantifying the signal strength of the Wnt signaling reporter mCherry along the fin ray from the tip of the regenerate down to the amputation plane in the same Tg(*7xTCF-XIa.Siam*:nlsmCherry)^ia5^ zebrafish (22). This allowed the determination of a Wnt-active zone (a region at the fin tip displaying relatively high mCherry expression) in these zebrafish, in addition to assessing signal intensity along the proximo-distal axis of the regrowing tissue (**Figures 2**, **3**). Importantly, Wnt signaling activity correlates with proliferation rates in regenerating zebrafish fins (17). In all fins, no matter the treatment, mCherry signal strength decreased over time, likely reflecting decreased proliferation rates occurring towards completion of regeneration. In comparison to the DMSO control, treatment with prednisolone resulted in a reduced Wnt-reporter signal (lower maximum fluorescence intensity) as well as a shorter Wnt-active zone (see boxes and arrows in **Figure 2**) at 9 dpa. At 14 dpa, Wnt signaling strength (maximum fluorescence intensity) in both treatment groups was similar, however, a reduction in the length of the Wnt-active zone in the prednisolone-treated zebrafish remained. This difference diminished over time, as the Wnt-active zone length reduced in size up to 21 dpa. Discontinuation of prednisolone treatment, as well as short-term prednisolone treatment in the late regeneration phase showed similar effects, resulting in modest suppression of Wnt activity at 21 dpa.

**Figure 4:**
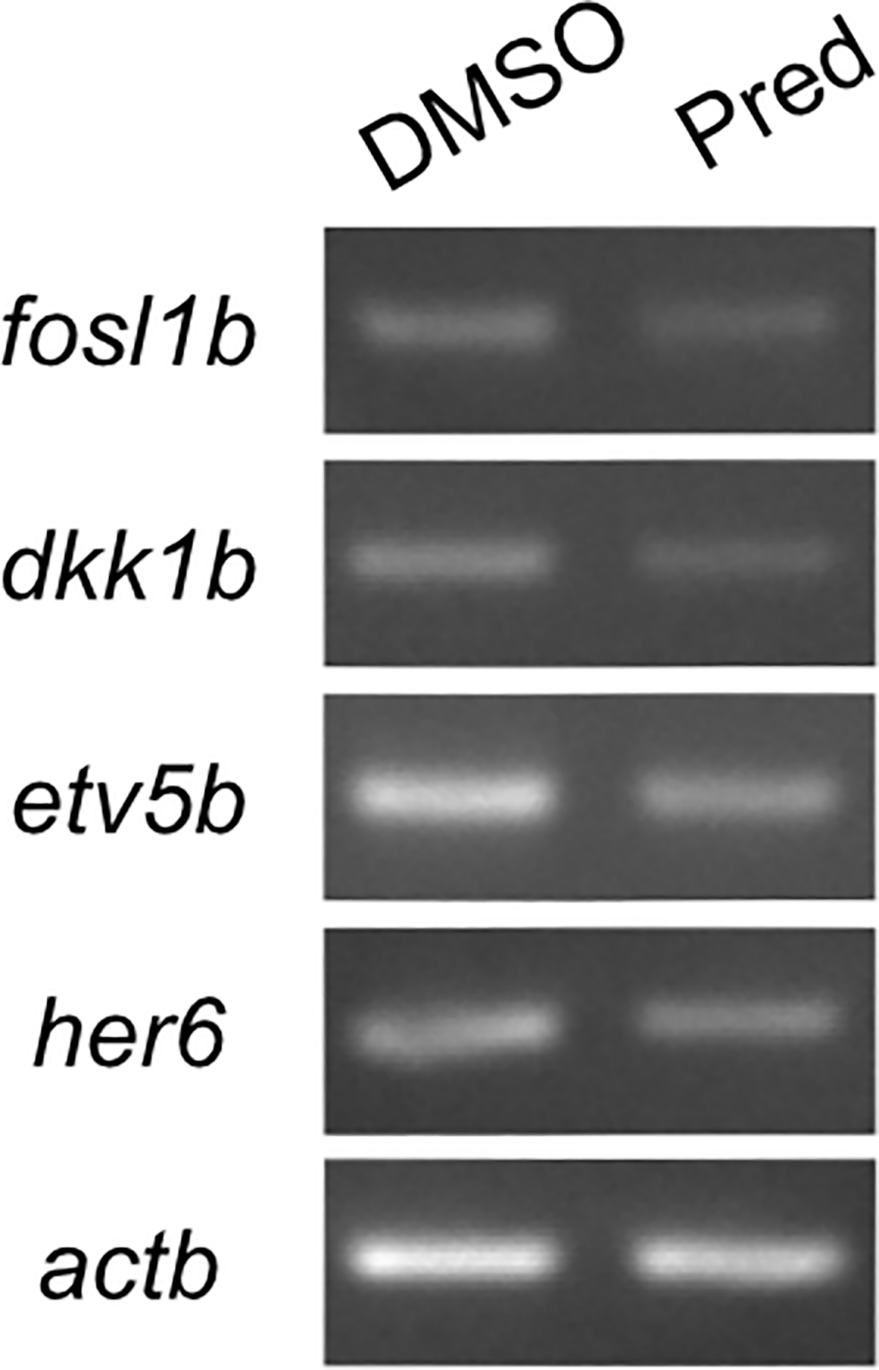
RT-PCR on Wnt, Fgf and Notch target genes and the Wnt signaling inhibitor *dkk1b*. 7-day prednisolone treatment after fin amputation reveals suppression of *fosl1b* (Wnt target gene), *etv5b* (*ETS variant transcription factor 5b*, Fgf target gene) and *her6* (*hairy-related 6*, Notch target gene) in fin regenerates. Likewise, *dkk1b* levels are reduced.

In order to confirm that Wnt signaling was affected by GC treatment, we performed RT-PCR on prednisolone and control treated regenerating fins. Indeed, *fosl1b (FOS like 1, AP-1 transcription factor subunit b)*, an orthologue to the human Wnt target *FOSL1* (30) was expressed at reduced levels after prednisolone treatment, as were targets of other signaling pathways (**Figure 4**). This supports the hypothesis that Wnt signaling is reduced upon high GC exposure.

### 3.2 GC treatment leaves cell proliferation in homeostatic skull bone, scales and brain tissue unaffected

**Figure 5:**
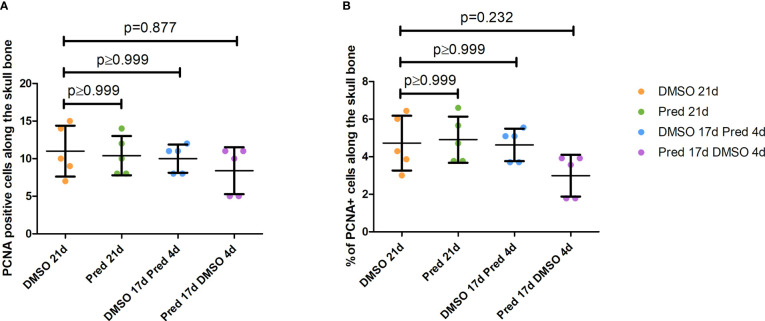
Quantification of PCNA positive cells along the skull bone of the zebrafish. **(A)** Total number of PCNA+ cells lining the skull bone. **(B)** Percentage of PCNA+ cells lining the skull bone (normalized to all cells lining the skull bone). Data are mean ± SD of PCNA positive cells lining bone in the different groups (21 days of DMSO, 21 days of prednisolone, 17 days of DMSO followed by 4 days of prednisolone and 17 days of prednisolone followed by 4 days of DMSO treatment). Each dot represents one biological replicate. Non-parametric testing because of non-normal distribution of the data. Statistical significance was tested by *post-hoc* Dunn’s multiple comparison after Kruskal-Wallis test. n=5 (4 females, 1 male in DMSO 21d, DMSO 17d Pred 4d, Pred 17 d DMSO 4d; 5 females in Pred 21d) in all groups with 1 section per individual.

**Figure 6:**
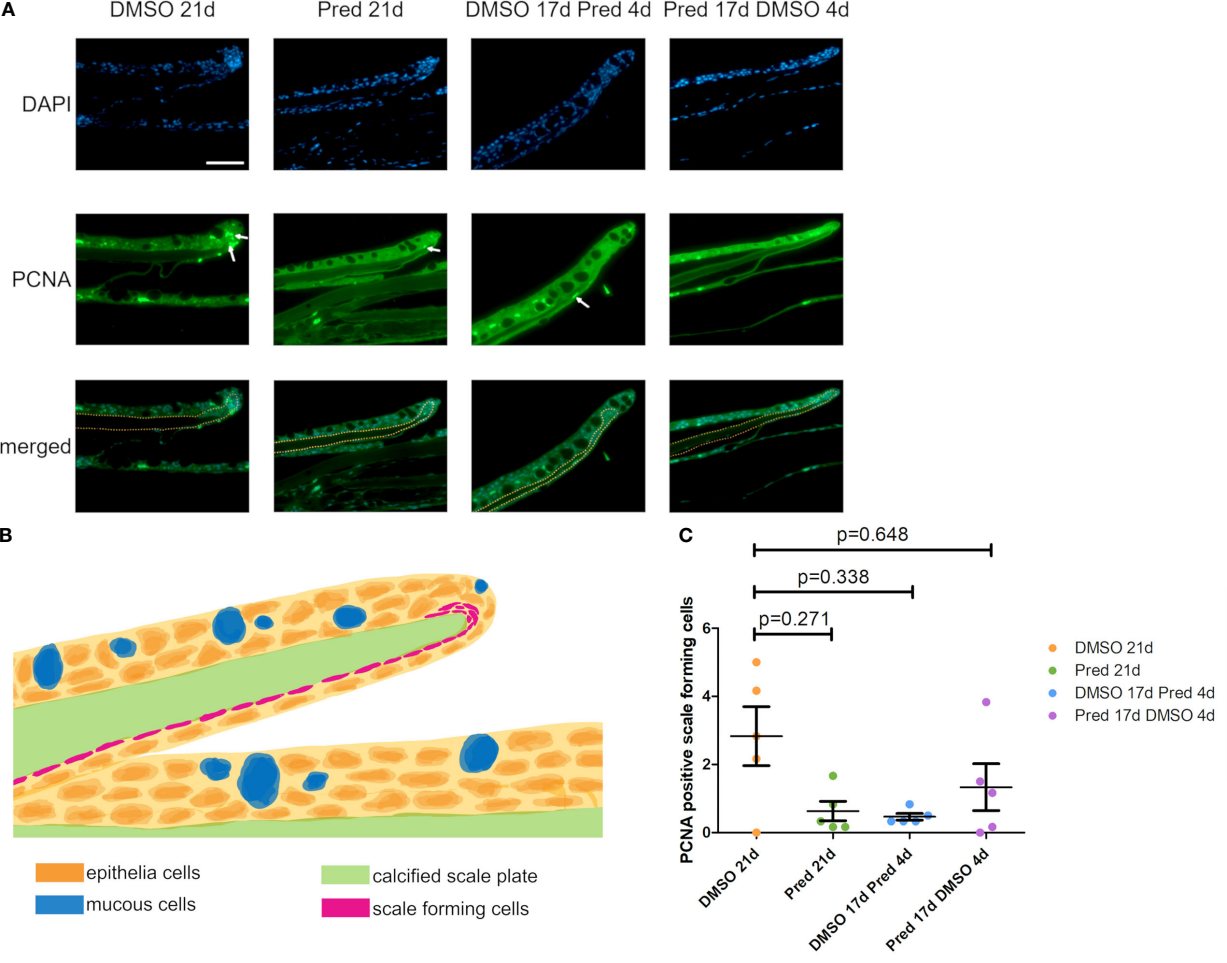
Proliferation of scale forming cells after treatment. **(A)** Immunohistochemical staining against PCNA and nuclear counterstain with DAPI. From left to right: fish underwent 21 days of DMSO, 21 days of prednisolone, 17 days of DMSO followed by 4 days of prednisolone and 17 days of prednisolone followed by 4 days of DMSO treatment. Scalebar 50 µm. White arrows indicate some PCNA+ cells. Yellow dotted lines outline the calcified scale plate together with the scale forming cells. **(B)** Schematic section view of a zebrafish scale in the zebrafish trunk. The calcified scale plate (green) is enclosed by the skin epithelium containing layers of epithelial (orange) and mucous cells (blue). The scale-forming cells (magenta) are in close proximity to the calcified scale plate. They cover the lower side of the scale and are located in higher number at the scale tip (in the marginal zone). **(C)** Quantification of PCNA+ scale forming cells in the different groups (21 days of DMSO, 21 days of prednisolone, 17 days of DMSO followed by 4 days of prednisolone and 17 days of prednisolone followed by 4 days of DMSO treatment). Data are mean ± SD of PCNA+ scale forming cells. Each dot represents one biological replicate. Non-parametric testing because of non-normal distribution of the data. Statistical significance was tested by *post-hoc* Dunn’s multiple comparison after Kruskal-Wallis test. n=5 (4 females, 1 male in DMSO 21d, DMSO 17d Pred 4d, Pred 17 d DMSO 4d; 5 females in Pred 21d) in all groups with 1 section per individual.

**Figure 7:**
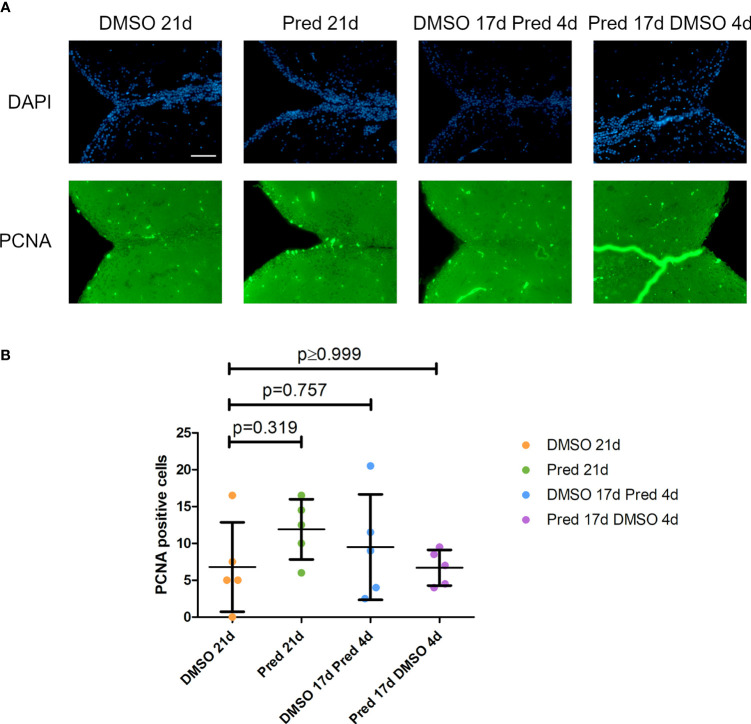
Cell proliferation in the telencephalon of zebrafish. **(A)** Immunohistochemical staining against PCNA and nuclear counterstain with DAPI. From left to right: fish underwent 21 days of DMSO, 21 days of prednisolone, 17 days of DMSO followed by 4 days of prednisolone and 17 days of prednisolone followed by 4 days of DMSO treatment. Scalebar 50 µm. **(B)** Quantification of PCNA+ cells in the telencephalon. Data are mean ± SD of PCNA+ cells in the different groups (21 days of DMSO, 21 days of prednisolone, 17 days of DMSO followed by 4 days of prednisolone and 17 days of prednisolone followed by 4 days of DMSO. Each dot represents one biological replicate. Parametric testing because of normal distribution of the data. Statistical significance was tested by *post-hoc* Dunnett’s multiple comparison after one-way ANOVA. n=5 (4 females, 1 male in DMSO 21d, DMSO 17d Pred 4d, Pred 17 d DMSO 4d; 5 females in Pred 21d) in all groups with 2 sections per individual.

The fact that prednisolone treatment affected regrowth and Wnt signaling as well as osteoblast proliferation during fin regeneration, as reported previously (11), led us to investigate whether proliferation of osteoblasts was altered in homeostatic conditions. Proliferation in cells along the skull bone was generally low, and there was no significant reduction of PCNA staining in these cells (**Figures 5A, B**). Scales were used as a second readout for bone matrix forming cell proliferation. Cells lining scale matrix and cells being located in the growth zone of the scale did not show any significant reduction in PCNA staining (**Figure 6**). Another tissue, which was unaffected by prednisolone treatment was the brain (telencephalon) which did not show any significant changes in proliferation (**Figures 7A, B**).

### 3.3 GC treatment decreases cell proliferation in the skin and intestine, as well as Wnt signaling in the skin

**Figure 8:**
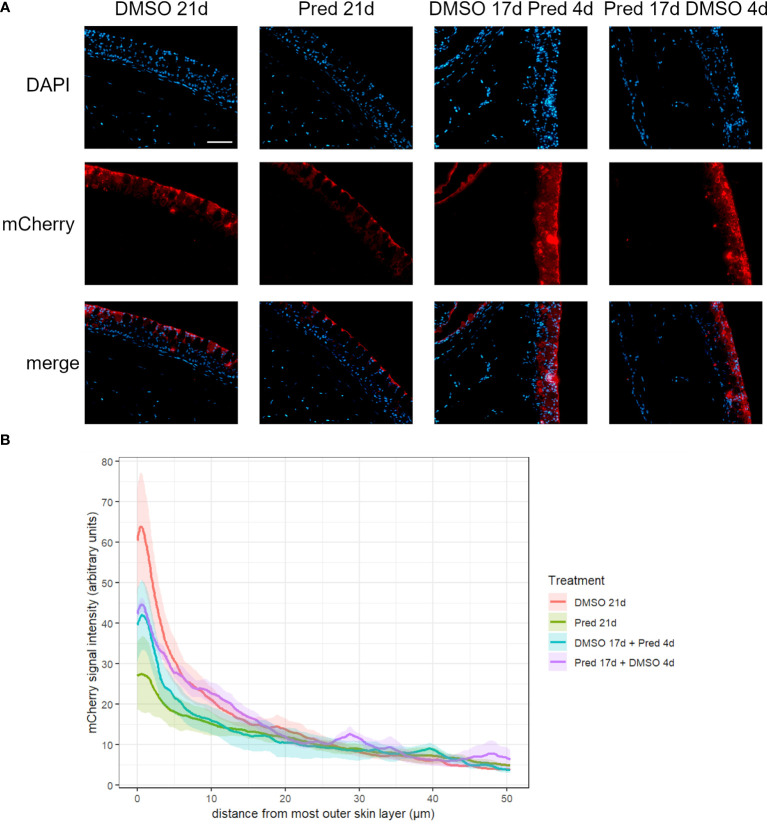
Wnt signaling in the skin of zebrafish. **(A)** Immunohistochemical staining against the Wnt-reporter mCherry and nuclear counterstain with DAPI. From left to right: fish underwent 21 days of DMSO, 21 days of prednisolone, 17 days of DMSO followed by 4 days of prednisolone and 17 days of prednisolone followed by 4 days of DMSO treatment. Scalebar 50 µm. **(B)** Quantification of the *7xTCF-Xia.Siam:*nlsmCherry Wnt reporter expression in the skin. Lines represent mean ± SEM of mCherry signal intensity for fish that underwent 21 days of DMSO, 21 days prednisolone, 17 days DMSO followed by 4 days prednisolone and 17 days of prednisolone followed by 4 days of DMSO treatment. Signal was measured from the outermost layer towards the inner layers of the skin. Average skin thickness is around 50 µm. n=5 (4 females, 1 male in DMSO 21d, DMSO 17d Pred 4d, Pred 17 d DMSO 4d; 5 females in Pred 21d) in all groups with 2 sections per individual.

**Figure 9:**
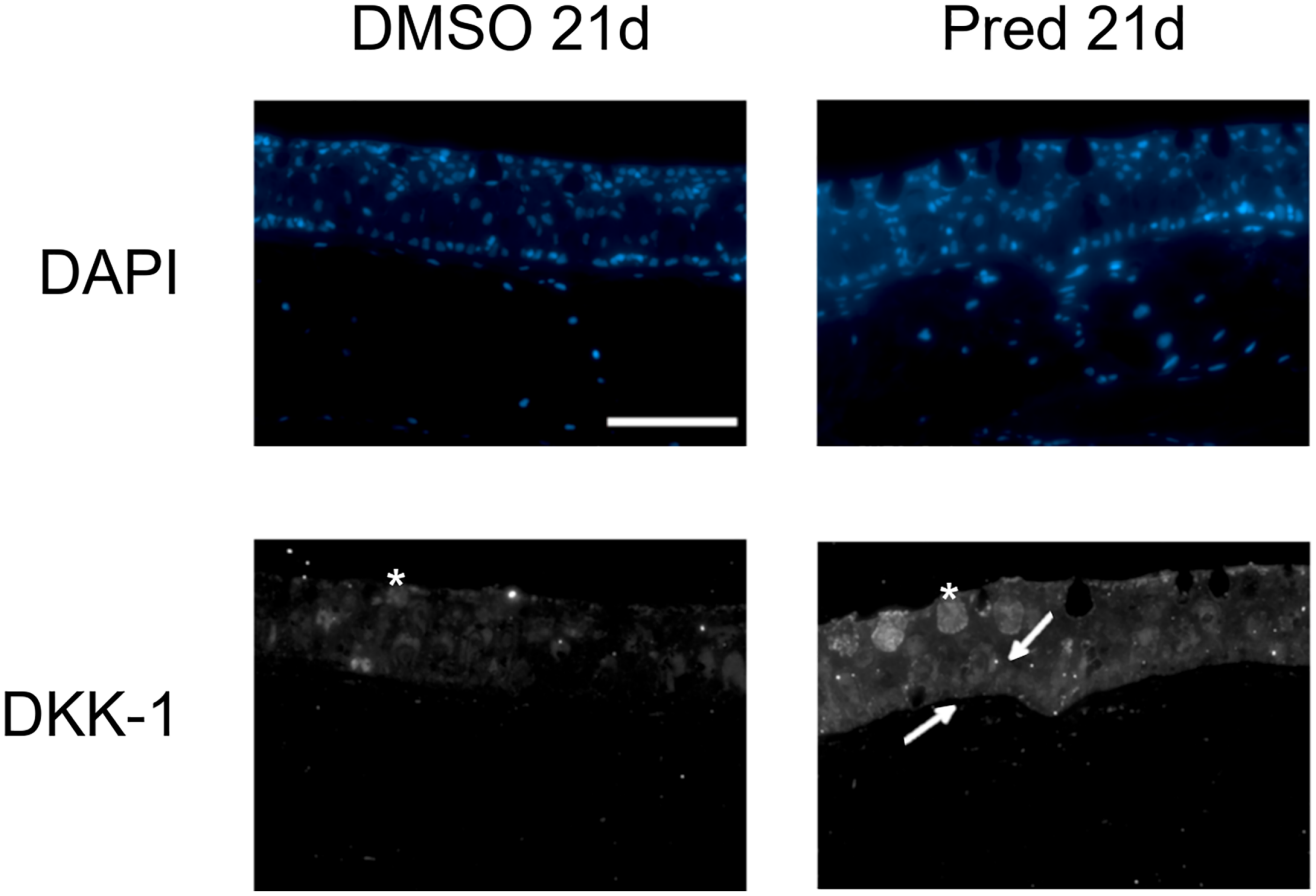
Dkk1 expression in the skin of zebrafish. Immunohistochemical staining against Dkk1 and nuclear counterstain with DAPI in zebrafish that underwent 21 days of DMSO or 21 days of prednisolone treatment. 4 out of 5 zebrafish with prednisolone treatment showed stronger staining in the basal portion of skin than DMSO treated zebrafish (4 out of 5 DMSO treated zebrafish with weak staining in the basal portion of the skin). Scale bar 50 µm. Arrows point to brighter signal in the basal portion of the skin. Asterisks indicate mucous cells. n=5 (4 females, 1 male in DMSO 21d, 5 females in Pred 21d) in both groups with 5 sections per individual.

**Figure 10:**
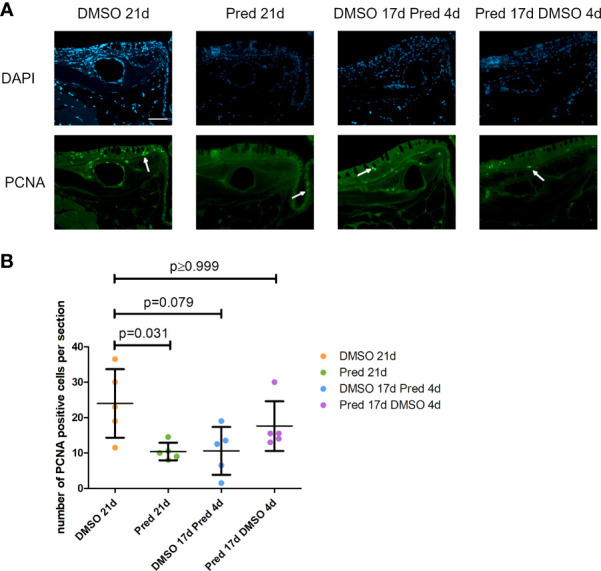
Cell proliferation in the skin of zebrafish. **(A)** Immunohistochemical staining against PCNA and nuclear counterstain with DAPI. From left to right: fish underwent 21 days of DMSO, 21 days of prednisolone, 17 days of DMSO followed by 4 days of prednisolone and 17 days of prednisolone followed by 4 days of DMSO treatment. Scalebar 50 µm. White arrows indicate PCNA+ cells. **(B)** Quantification of PCNA+ cells in the skin. Data are mean ± SD of PCNA+ cells per section for the treatment conditions 21 days of DMSO, 21 days of prednisolone, 17 days of DMSO followed by 4 days of prednisolone and 17 days of prednisolone followed by 4 days of DMSO. Each dot represents one biological replicate. Non-parametric testing because of non-normal distribution of the data. Statistical significance was tested by *post-hoc* Dunn’s multiple comparison after Kruskal-Wallis test. n=5 (4 females, 1 male in DMSO 21d, DMSO 17d Pred 4d, Pred 17 d DMSO 4d; 5 females in Pred 21d) in all groups with 2 sections per individual.

**Figure 11:**
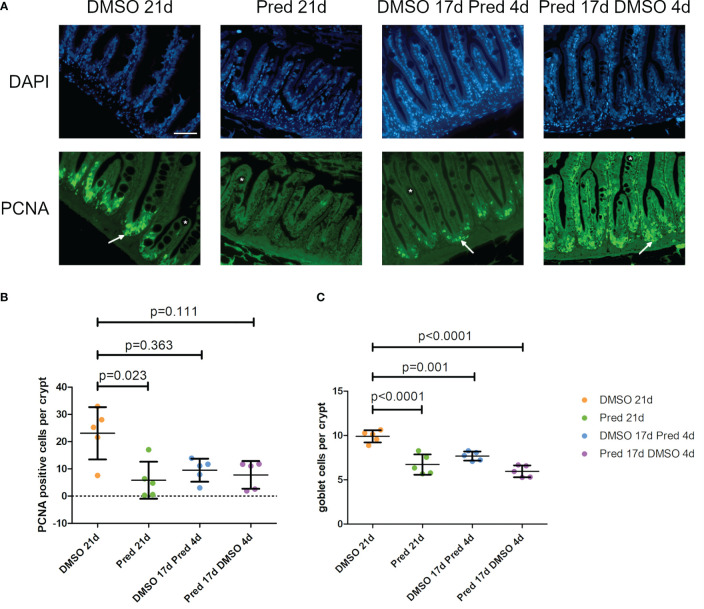
Cell proliferation and goblet cell number in the crypts of the intestine. **(A)** Immunohistochemical staining against PCNA and nuclear counterstain with DAPI. From left to right: fish underwent 21 days of DMSO, 21 days of prednisolone, 17 days of DMSO followed by 4 days of prednisolone and 17 days of prednisolone followed by 4 days of DMSO treatment. White asterisks indicate goblet cells and white arrows indicate PCNA+ cells in the crypts. Scalebar 50 µm. Sample “Pred 17d DMSO 4d” showed increased autofluorescence. **(B)** Quantification of PCNA+ cells per crypt in the intestine. Data are mean ± SD of PCNA+ cells per crypt for the treatment conditions 21 days of DMSO, 21 days of prednisolone, 17 days of DMSO followed by 4 days of prednisolone and 17 days of prednisolone followed by 4 days of DMSO. Each dot represents one biological replicate. Non-parametric testing because of non-normal distribution of the data. Statistical significance was tested by *post-hoc* Dunn’s multiple comparison after Kruskal-Wallis test. n=5 (4 females, 1 male in DMSO 21d, DMSO 17d Pred 4d, Pred 17 d DMSO 4d; 5 females in Pred 21d) in all groups with 3 sections per individual. **(C)** Quantification of goblet cells per intestinal crypt. Data are mean ± SD of goblet cells per crypt for the treatment conditions 21 days of DMSO, 21 days of prednisolone, 17 days of DMSO followed by 4 days of prednisolone and 17 days of prednisolone followed by 4 days of DMSO. Each dot represents one biological replicate. Parametric testing because of normal distribution of the data. Statistical significance was tested by *post-hoc* Dunnett’s multiple comparison after one-way ANOVA. n=5 (4 females, 1 male in DMSO 21d, DMSO 17d Pred 4d, Pred 17 d DMSO 4d; 5 females in Pred 21d) in all groups with 8 sections per individual.

**Figure 12:**
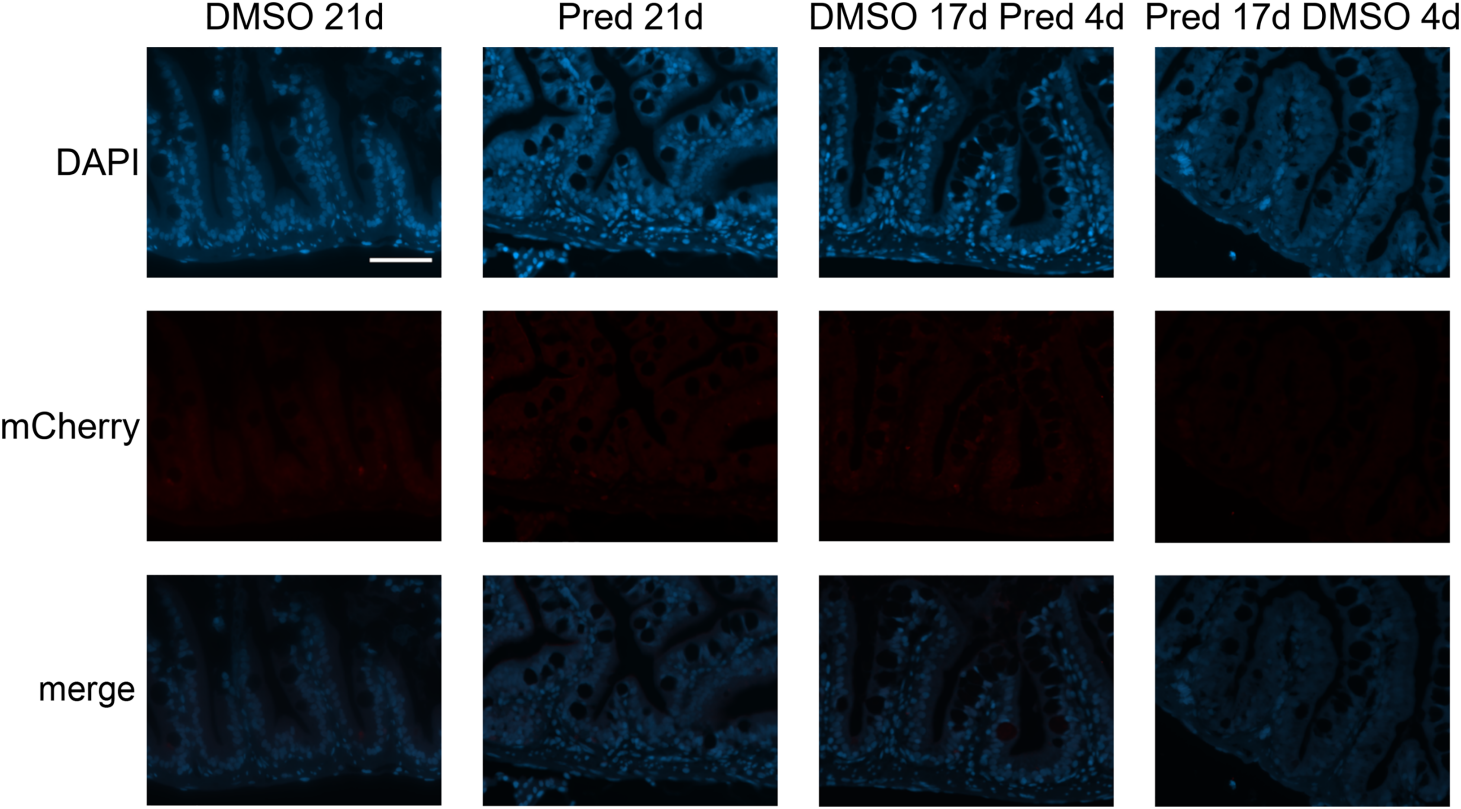
Absence of mCherry specific staining in Wnt-reporter *7xTCF-Xia.Siam*:nlsmCherry zebrafish intestine. Immunohistochemical staining against the Wnt-reporter mCherry and nuclear counterstain with DAPI. From left to right: fish underwent 21 days of DMSO, 21 days of prednisolone, 17 days of DMSO followed by 4 days of prednisolone and 17 days of prednisolone followed 4 days of DMSO treatment. With our staining method, we were unable to detect mCherry+ cells in the intestine of transgenic reporter zebrafish. Scalebar 50 µm. n=5 (4 females, 1 male in DMSO 21d, DMSO 17d Pred 4d, Pred 17 d DMSO 4d; 5 females in Pred 21d) in all groups with 3 sections per individual.

To assess whether the effects of prednisolone on Wnt signaling and tissue growth in the fin reflect the status of signaling pathway activity and potentially tissue turnover in highly proliferative tissues, immunohistochemical staining was performed against mCherry (Wnt signaling reporter) and PCNA in the skin and intestine of treated zebrafish. Furthermore, Dkk1, a Wnt inhibitor, was detected by immunohistochemistry in skin tissue. Changes in cell proliferation could be observed in the skin and intestine, along with the respective changes in Wnt signaling reporter activity and increased Dkk1 presence in the skin (**Figures 8–11**). Wnt signaling reporter staining was undetectable in the intestine (**Figure 12**), pointing at either lacking expression or too low-level expression of the reporter to be detected with the used immunohistochemistry protocol. 21 days of prednisolone treatment resulted in a strong decrease of Wnt signaling in the skin compared to the DMSO control (**Figure 8A**). Prednisolone treatment led to a reduction of the reporter signal to less than half of the control in the outermost layer of the skin (**Figure 8B**). In line with this, basal skin tissue showed more prominent expression of Dkk1 in prednisolone treated zebrafish (**Figure 9**). Of note, Dkk1 protein was also detected in skin mucous cells of our samples (asterisks in **Figure 9**). Discontinuation of the treatment during the last four days partly reversed the effect of suppressed Wnt reporter activity in the skin. Importantly, treatment with prednisolone during only the last four days of the experiment resulted in a similar level of Wnt signaling as was seen in zebrafish which had experienced a 17-day treatment with prednisolone followed by a 4-day recovery period. These results correlate with our findings of suppressed Wnt signaling in fin regeneration.

Analyses of PCNA+ cells in the skin showed a significant reduction in cell proliferation after 21-day prednisolone treatment (average of 10 ± 2.5 PCNA+ cells) in comparison to the DMSO control with an average of 24 ± 9.68 PCNA+ cells per skin section (**Figures 10A, B**). On the other hand, a stop of prednisolone treatment during the last four days resulted in no significant reduction in the PCNA+ cell number (average of 18 ± 7.01 PCNA+ cells). Similarly, skin proliferation was not significantly decreased by a 4-day prednisolone treatment (average of 11 ± 6.8 PCNA+ cells).

Analysis of PCNA+ cells in the crypts of the intestine revealed reduced cell proliferation in samples treated with prednisolone for 21 days (**Figures 11A, B**). On average, only 6 ± 6.8 cells per crypt were PCNA+ after 21-day prednisolone treatment as compared to an average of 23 ± 9.6 PCNA+ cells after 21 days of DMSO treatment. Discontinued prednisolone treatment did not significantly impact crypt cell proliferation (8 ± 5.1 PCNA+ cells) in comparison to the DMSO control; similarly, PCNA+ cell number was not significantly altered by short-term prednisolone treatment (10 ± 4.2 PCNA+ cells). In contrast, assessment of the number of goblet cells per crypt revealed their significant reduction in all prednisolone treatment conditions (**Figures 11A, C**). While the control treatment with DMSO showed an average of 10 ± 0.69 goblet cells per crypt, the number of goblet cells was reduced to an average of 7 ± 1.14 by 21-day treatment with prednisolone, 8 ± 0.52 by 17-day prednisolone + 4-day DMSO treatment and 6 ± 0.67 goblet cells by short-term prednisolone treatment.

### 3.4 GC treatment reduces leukocytes in the intestine

**Figure 13:**
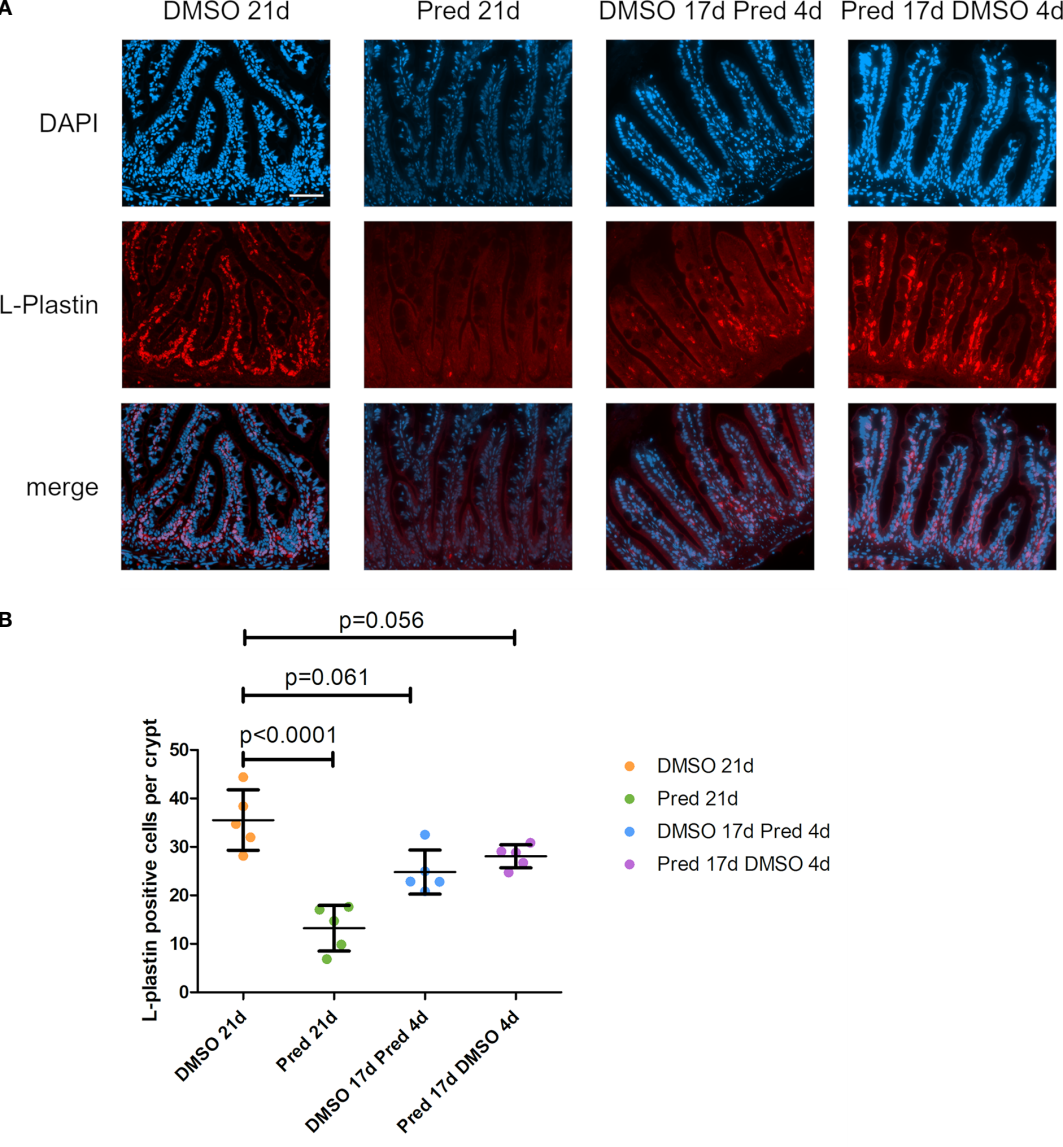
Leukocytes in the zebrafish intestine. **(A)** Immunohistochemical staining against L-Plastin and nuclear counterstain with DAPI. From left to right: fish underwent 21 days of DMSO, 21 days of prednisolone, 17 days of DMSO followed by 4 days of prednisolone and 17 days of prednisolone followed by 4 days of DMSO treatment. Scalebar 50 µm. **(B)** Quantification of L-Plastin+ cells per crypt of the zebrafish intestine. Data are mean ± SD of L-Plastin+ cells in the 4 groups (21 days of DMSO, 21 days of prednisolone, 17 days of DMSO followed by 4 days of prednisolone and 17 days of prednisolone followed by 4 days of DMSO). Each dot represents one biological replicate. Parametric testing because of normal distribution of the data. Statistical significance was tested by *post-hoc* Dunnett’s multiple comparison after one-way ANOVA. n=5 (4 females, 1 male in DMSO 21d, DMSO 17d Pred 4d, Pred 17 d DMSO 4d; 5 females in Pred 21d) in all groups with 5 sections per individual.

L-plastin is a cross-linking protein for actin filaments, specifically found in the cytosol of leukocytes (31). To investigate the influence of prednisolone on immune cells in the intestine the number of L-plastin+ cells per crypt was assessed as a readout for leukocytes (**Figures 13A, B**). A significant reduction of leukocytes was observed after 21 days of prednisolone treatment. While DMSO treatment for 21 days resulted in an average of 36 ± 6.2 L-plastin+ cells per crypt, prednisolone treatment resulted in an average of 13 ± 4.7 L-plastin+ cells. Discontinuation of prednisolone treatment after 17 days (4-day recovery) saved the intestine from a significant reduction of L-plastin+ cell number compared to DMSO treatment, with an average of 28 ± 2.37 L-plastin+ cells, suggesting reversibility of leukocyte suppression. Likewise, 4-day prednisolone treatment did not significantly suppress leukocyte number in the intestine, indicating that longer treatment is required to reduce intestinal leukocyte number.

## 4 Discussion

Here, we studied the effects of GC treatment in zebrafish, a teleost species which shows life-long proliferation capacity in a variety of tissues such as the fin which grows at the fin tips (32). After injury, proliferation capacity is strongly boosted during regeneration of the fin and many other organs (33), allowing for the distinction between low-level tissue proliferation during adult growth and tissue homeostasis and regenerative settings characterized by much higher proliferation rates and induction of pro-regenerative signaling cascades (34). Therefore, we assessed the impact of the GC prednisolone on Wnt signaling and cell proliferation in different homeostatic zebrafish tissues as well as regenerating fin tissue. We also investigated the potential recovery of a 17-day prednisolone treatment and the impact of short-term treatment with prednisolone.

Assessment of regenerate length and Wnt reporter signal in the growing fin was used to determine the effect of prednisolone on growth mirroring proliferation during regeneration. As shown previously, regeneration and cell proliferation in the fin are negatively affected by prednisolone treatment (11). Furthermore, Wnt signaling is known as a mitogenic pathway in numerous tissues (35) allowing for high rates of proliferation. Treatment with prednisolone for 21 days after amputation diminished fin regenerate length significantly, both in a previous study (11) and the study at hand. Likewise, treatment with the GC dexamethasone exerted an inhibitory effect on fin regrowth and brain regeneration (36, 37). Here, we show that prednisolone treatment results in a reduction of the Wnt-active proliferation zone of fin regenerates. A study focusing on human neural progenitor cells in cell culture demonstrated that dexamethasone induces upregulation of the Wnt signaling inhibitor DICKKOPF1 (DKK1) (38). This upregulation is due to increased transcription of *DKK1* mRNA through the action of dexamethasone, in a dose- and time-dependent manner, which was also shown in primary cultured human osteoblasts (38, 39). This effect could be circumvented by adding the GR antagonist mifepristone (38, 40). The inhibitory effect of GCs on Wnt signaling is in line with our findings of suppressed Wnt reporter gene expression in the growth zone of the regenerate along with the observed reduced expression of *fosl1b* in prednisolone treated fin regenerates. The observed reduction in Wnt signaling (along with changes in Fgf and Notch target gene expression, see **Figure 4**) indicates that proliferation could no longer be sustained, which resulted in shorter growth of the fin regenerates.

Immunohistochemistry revealed negative effects of prednisolone on cell proliferation and Wnt signaling in the skin and intestine. First, 21 days of prednisolone treatment reduced Wnt signaling in the skin compared to the control. Previous reports have shown that GCs lead to a thinning of the epidermis by inhibiting the proliferation and migration of keratinocytes (41). Furthermore, Wnt signaling is known to play an important role in tissue homeostasis and stem cell activation of the skin (42). For example, aberrations in Wnt signaling were found to be essential for the initiation and progression of keratinocyte carcinoma by increasing tumor cell proliferation (42). This leads to the assumption that GCs might exert their negative effects on the epidermis at least partly by influencing Wnt signaling. In line with this, GC treatment increased the presence of Dkk1 in the basal part of the skin, indicating that GCs suppress Wnt signaling in the skin by increasing Dkk1 expression in zebrafish.

Second, homeostatic tissue turnover in the intestine and skin was negatively affected by prednisolone treatment. 21-day prednisolone treatment significantly reduced the number of proliferating cells in the intestinal crypts compared to the control. Similarly, significant reduction of skin cell proliferation was only observed after 21-day treatment, indicating a recovery potential of the skin and intestine which both did not show any significant impairment of proliferation in the remaining test groups. Notably, formation of goblet cells per crypts was reduced in all conditions of prednisolone treatment. During undisturbed mammalian homeostasis, these cells are continuously renewed every three to five days from the highly proliferative stem cells at the base of the crypts (43). Both reduction of proliferation and impaired differentiation might contribute to decreased goblet cell number in excess GC conditions. Moreover, in line with its immunosuppressive function, prednisolone treatment significantly lowered the cell number of intestinal leukocytes; however only if administered for a sufficient amount of time. Leukocyte reduction is likely caused by GC-induced apoptosis of lymphocytes (44), at least partially, and might be involved in the antiproliferative effect which prednisolone exerts on the intestinal tissue. If so, this would be in line with the known importance of immune cells during tissue zebrafish regeneration as cells supporting stem and progenitor cell proliferation and subsequent differentiation (45, 46).

Notably, we did not detect a clear Wnt signaling reporter activity in the zebrafish intestine. This may indicate that cell turnover in zebrafish crypts does not strongly depend on Wnt signaling; alternatively, there might be issues of Wnt signaling reporter expression in the intestine or with our staining protocol. Peron et al. (47) reported that Signal transducer and activator of transcription 3 (Stat3) is expressed in a Wnt-dependent fashion in intestinal crypts in zebrafish (47) which points to the relevance of Wnt signaling in zebrafish gut homeostasis. To resolve this discrepancy, staining for Wnt signaling components should be performed in the future, in particular, because a potential interaction of the GR with STAT3 was already described (48, 49). Moreover, a report on the expression of IL-10 in human B cells suggests an induction of substantial STAT3 by GC *via* direct interaction of the GR with STAT3 (50).

The maintenance of stem cells and differentiation into specific cell lineages, like the goblet cells, is orchestrated by a complex interplay of multiple pathways, not only the Wnt/β-catenin pathway. This also includes pathways such as PI3-kinase/Akt and Notch signaling (51, 52). Taken together, we observed a correlative impairment of Wnt signaling and cell proliferation due to prednisolone treatment in homeostatic skin, a reduction of proliferation in the intestinal crypts, as well as suppressed Wnt signaling and diminished growth under regenerative conditions in the fin. Of note, we did not detect increased *dkk1b* levels in regenerating fins treated with prednisolone for 7 days by RT-PCR (**Figure 4**), indicating that other mechanisms than enhanced *dkk1b* expression drive Wnt suppression during fin regeneration. Despite these tissue-specific differences of Wnt suppression mechanisms, our study indicates that the status of regenerating fins, which are convenient to study due to their accessibility and transparency, can serve as a first approximation to the status of other proliferative tissues (the skin and intestine), and that proliferation and Wnt signaling are both under control of the stress response in these tissues. It remains to be tested whether the anti-proliferative effects of prednisolone in the mentioned tissues are downstream of suppressed Wnt signaling or whether both, Wnt inhibition and suppression of proliferation, occur independently of each other. However, a study on human osteosarcoma cells shows that the GR represses cyclin D1, the function of which is required for cell cycle G1/S transition, by targeting Tcf-β-catenin, thereby providing evidence for a direct link between GC and Wnt signaling in cell cycle repression by GR (53). This is supported by findings in the osteoblast-like cells MC3T3-E1, which show that the inhibitory effect of GCs on the cell cycle during osteoblast differentiation is mediated both in a Wnt-independent manner and by abrogation of Wnt signaling, which involves actions up- and downstream of GSK3β. Both mechanisms employed by GCs contribute to the attenuation of the G1-S cell cycle transition by suppression of the LEF/TCF transcriptional activity (54).

Third, we hypothesized that discontinuation of prednisolone treatment after 17 days and a recovery period of 4 days would be sufficient for a partial reversal of the prednisolone induced effects. This generally seems to be the case, as suppression of fin regeneration, proliferation, L-plastin+ cell infiltration, and Wnt signaling reporter activity in the skin were not as pronounced in case prednisolone treatment was either discontinued or lasted only a few days. Our data indicate that a 4-day treatment at the given dose is not sufficient to suppress these parameters in the studied tissues. A remarkable exception to this were the intestinal goblet cells whose number significantly decreased in all prednisolone treatment conditions, which suggests that their function is extremely sensitive to increased GC levels. In case of the skin, it is likely that the minimum time to achieve significant suppression of Wnt signaling would correlate with the time which is needed for fin regeneration to be significantly impaired, which is 7 days of treatment (11). In this regard, it will be interesting to further compare the effects of short and sustained GC treatment (and recovery thereof) in fin regeneration and tissue turnover of highly proliferative tissues.

Last but not least, we show here that treatment with prednisolone did not affect uninjured, homeostatic zebrafish bone in terms of bone forming cell proliferation. Proliferation of cells lining bone matrix was overall low and not further reduced by GC treatment as shown in skull bone and homeostatic scales. Similarly, parts of the brain did not change the number of proliferating cells, opposite to the observed effects in the skin, fin, and intestine. Both tissue types (bone and brain) exhibit low levels of proliferation in uninjured conditions. Thus, our observations agree with a previous study which suggested that GCs mainly exert their negative effects in highly proliferative tissues (11). Studies on methylprednisolone observed its ability to cross the blood-brain barrier, however just at a low rate (55). Therefore, caution is warranted as the actual concentration of prednisolone in the brain of treated zebrafish is not known in our experiments. Further experiments are needed to test the effects of prednisolone treatment on proliferation in brain tissue. Of note, brain-injured zebrafish show decreased levels of stem cell proliferation upon treatment with dexamethasone, a more potent GC, an effect that is linked to immunosuppression (36).

Taken together, we identified a dampening effect of prednisolone on Wnt signaling and proliferation in highly proliferative tissues in homeostasis and regeneration. Our study raises interesting questions for future research: What is the minimal time which is required for the intestine to recover goblet cell number? Do individuals treated with GC show intestinal barrier dysfunction? Do longer treatments with GCs affect proliferation of stem and progenitor cells in the brain (36)? How does GC treatment influence other pathways that are involved in intestinal proliferation and differentiation besides Wnt signaling, such as Notch- and Fgf-signaling? Does prednisolone affect Wnt signaling in fin and skin tissue directly, or does immunosuppression play a role? Addressing these questions will increase our understanding of the adverse effects of GCs which may help to reduce their impact in the future. Furthermore, this and future studies will also underscore the use of the fin regeneration paradigm to study adverse effects of frequently prescribed drugs in tissues with high cell turnover.

## Data availability statement

The original contributions presented in the study are included in the article/supplementary material. Further inquiries can be directed to the corresponding author.

## Ethics statement

The animal study was reviewed and approved by Landesdirektion Sachsen.

## Author contributions

Experiments were designed by FK, performed and analyzed by LF, ACL-D, KG, and FK. LF, ACL-D and FK interpreted data and all authors edited the manuscript. LF wrote the first draft of the manuscript. LF and FK accept responsibility for the integrity of data analysis. All authors contributed to the article and approved the submitted version.
